# Redescription of the Siamese shield leech *Placobdelloides siamensis* with new host species and geographic range

**DOI:** 10.1051/parasite/2018056

**Published:** 2018-11-26

**Authors:** Krittiya Chiangkul, Poramad Trivalairat, Watchariya Purivirojkul

**Affiliations:** 1 Animal Systematics and Ecology Speciality Research Unit, Department of Zoology, Faculty of Science, Kasetsart University 50 Ngam Wong Wan Road Chatuchak Bangkok 10900 Thailand

**Keywords:** *Placobdelloides*, Glossiphoniidae, Hirudinea, Clitellata, Leech, Snail-eating turtle, *Malayemys*, Bangkok, Thailand

## Abstract

The Siamese shield leech *Placobdelloides siamensis* (Oka, 1917) Sawyer, 1986 (Euhirudinea: Glossiphoniidae) was collected from five new host species, Southeastern Asian Box Turtle (*Cuora amboinensis*), Yellow-headed Temple Turtle (*Heosemys annandalii*), Malayan Snail-eating Turtle (*Malayemys macrocephala*), Mekong Snail-eating Turtle (*M. subtrijuga*), and Khorat Snail-eating Turtle (*M. khoratensis*) and was found for the first time in Udon Thani, Thailand. Examination of live leeches provided, for the first time, data on coloration and the combination of parental care behavior, both carrying cocoons and attaching cocoons to the substrate. This species was separated from its congeners based on the following characters: one pair of eyes; spines at proboscis subterminal; mouth terminal on oral sucker; absent plaque in neck region; gonopores located in furrow and separated by two annuli; distinctly triannulated mid-body segments; crop with seven pairs and branched caeca; caudal sucker slightly over half of maximum body width; and strongly dorsal papillae. Phylogenetic relationships based on the COI and ND1 genes were clarified and demonstrated that the species is distinct from others. The original description was amended and the taxonomic history is discussed.

## Abstract (fr)

La sangsue *Placobdelloides siamensis* (Oka, 1917) Sawyer, 1986 (Euhirudinea: Glossiphoniidae) a été collectée chez cinq nouvelles espèces d’hôtes, les tortues *Cuora amboinensis*, *Heosemys annandalii*, *Malayemys macrocephala*, *M. subtrijuga* et *M. khoratensis*, et a été trouvée pour la première fois à Udon Thani, en Thaïlande. L’examen des sangsues vivantes a fourni pour la première fois des données sur la coloration et la combinaison du comportement des parents en matière de protection parentale, qui à la fois portent des cocons et les fixent au substrat. Cette espèce a été séparée de ses congénères selon les caractères suivants: une paire d’yeux ; épines au proboscis subterminal ; bouche terminale sur la ventouse orale ; plaque absente dans la région du cou ; gonopores situés dans un sillon et séparés par deux anneaux ; segments du milieu du corps distinctement triannulés ; jabot avec sept paires de caeca ramifiés ; ventouse caudale légèrement au-dessus de la moitié de la largeur maximale du corps ; et papilles fortement dorsales. Les relations phylogénétiques basées sur les gènes COI et ND1 ont été clarifiées et ont démontré que l’espèce est distincte des autres. La description originale a été modifiée et l’histoire taxonomique est discutée.

## Introduction

*Placobdelloides* Sawyer, 1986 [20] is a genus of glossiphoniid leeches, currently composed of 16 species found worldwide, *i.e.*, in Africa (*Placobdelloides fimbriata* Johansson, 1909 [6]; *P. jaegerskioeldi* Johansson, 1909; *P. multistriata* Johansson, 1909 [13, 19]), Australia and the United States, eastward to India (*P. fulva* Harding, 1921 [17]; *P. emydae* Harding, 1924 [4, 10]; *P. undulata* Harding, 1924 [11]; *P. horai* Baugh, 1960 [2]; *P. indica* Baugh, 1960 [2]), and Southeast Asia (*P. siamensis* Oka, 1917 in China and Thailand [1]; *P. okadai* Oka, 1925 [18] in China; *P. okai* Soós, 1969 [22]; *P. stellapapillosa* Goverdich *et al*., 2002 [9] in Malaysia and Singapore), and throughout Australia and New Zealand (*P. octostriata* Grube, 1866 [24]; *P. maorica* Benham, 1907 [15]; *P. bancrofti* Best, 1931 [16]; *P. bdellae* Ingram, 1957 [12]). Lacking a jaw, they usually feed with a protrusible proboscis and are predacious or sanguivorous, or both, on a variety of prey living in nearby water systems, such as shrimps, waterfowl, fish, amphibians, turtles, crocodiles or mammals (*P. octostriata* Grube, 1866; *P. maorica* Benham, 1907; *P. bancrofti* Best, 1931; *P. bdellae* Ingram, 1947) [9, 17, 20, 21, 24].

In Thailand, turtles are commonly used in the practice of religious merit release and are believed to grant a long life to the releaser. Frequently found species of turtles include Southeast Asian Box Turtle (*Cuora amboinensis* Daudin, 1802), Yellow-headed Temple Turtle (*Heosemys annandalii* Boulenger, 1903), Black Marsh Turtle (*Siebenrockiella crassicollis* Gray, 1831) and Red-eared Slider Turtle *Trachemys scripta elegans* Wied-Neuwied, 1839 (alien species). Snail-eating turtles are freshwater species belonging to the *Malayemys* genus, with three species members, *Malayemys macrocephala* Gray 1859, *M. subtrijuga* Schlegel and Müller 1845 and *M. khoratensis* Ihlow, Vamberger, Flecks, Hartmann, Cota, Makchai, Meewattana, Dawson, Kheng, Rödder and Fritz 2016, from Southeast Asia [3, 7, 23]. There have been almost no reports on their infection with leeches before; however, in this study, these turtles from various areas were found to be heavily infected with freshwater leeches, subsequently identified as *Placobdelloides siamensis*.

The Siamese shield leech *P. siamensis* was originally described in the early 20th century as *Hemiclepsis siamensis* Oka, 1917 from the black marsh turtle *Bellie crassicollis* Gray, 1831 (currently, *S. crassicollis*), which originated from Lampam, Patalung, Thailand, before having been reclassified to the current name by Sawyer in 1986 [1, 5, 20]. Even though Siddall *et al.* [21] have previously sequenced and reported this species from Wat Bovorn, Bangkok, Thailand, they did not describe the host species, nor did they update the data. Therefore, in this study, we examined information on *P. siamensis* to expand the original description by updating the known morphology and host range in Thailand, and including the ban on the release of turtles to prevent the spread of *P. siamensis.*

## Materials and methods

### Leech preparation

Snail-eating turtles (*Malayemys macrocephala* = 18; *M. Subtrijuga* = 12) and *Heosemys annandalii* were collected from ponds at Kasetsart University, Bangkok Province, Thailand (13°50′53.6ʺ N, 100°33′47.3ʺ E) from February to July 2017. Turtles were collected by hand at night. Some leeches were removed using forceps and then stored in 70% ethanol for identification, while others were stored in absolute ethanol.

### Morphological study

Approximately 3852 individual leeches were collected, from 20 host specimens, 9 *M. khoratensis* from Montri Sumontha (Udon Thani District, Udon Thani Province, 17°36′55.5ʺ N, 102°81′42.7ʺ E) and 11 *Cuora amboinensis* from the Phraya Suren Temple market, Bangkok Province (13°52′23.6ʺ N, 100°42′03.6ʺ E). For morphological characterization, leeches were examined for eye number and placement, annulation, digestive system (including the number and structure of gastric ceca), and reproductive system under a DVM6 digital microscope (Leica Microsystems (SEA) Pte. Ltd.) at a 2350× magnification. For scanning electron microscopy (SEM), leeches were preserved in absolute alcohol, then dried using the critical point dry (CPD) technique, coated in gold, and examined under a microscope.

### Molecular analysis

Leeches were cut into two pieces equally; the anterior part was kept in an absolute ethanol. Posterior part was used for DNA isolation with the DP304-02 TIANamp Genomic DNS, following the protocol given for the purification of total DNA from animal tissue (spin column). For the proteinase K treatment step, tissue samples were lysed for several hours at 56 °C. DNA was eluted from the spin column with 150 μL of buffer.

Polymerase chain reactions (PCRs) were prepared using EP0402 TAQ DNA Polymerase. Primers were ordered from Integrated DNA Technologies and were comprised of two primers each for cytochrome *c* oxidase subunit I (CO-I) and nicotinamide adenine dinucleotide dehydrogenase subunit I (ND-1), as specified by Light and Siddall (1999) [14]. Specifically, the CO-I primers were LCO1490 (5′–GGT CAA CAA ATC ATA AAG ATA TTG G–3′) and HCO2198 (5′–TAA ACT TCA GGG TGA CCA AAA AAT CA–3′). The ND-I primers were LND300 (5′–TGG CAG AGT AGT GCA TTA GG–3′) and HND1932 (5′–CCT CAG CAA AAT CAA ATG G–3′). Final volume of PCR reactions was 30 mL with 3 mL of leech genomic DNA added per reaction. DNA was amplified under the following PCR conditions: 94 °C for 5 min; 35 cycles of 94 °C for 30 s, 50°C for 30 s, 72 °C for 45 s; 72 °C for 7 min.

### Statistical analysis

PCR products were purified and sequenced using either the HCO2198 and LCO1490 primers for the CO-I products or the HND1932 and LND300 for the ND-I products by Macrogen Korea. The sequences obtained were submitted to GenBank (Table 1). Comparative sequences were retrieved from GenBank. The DNA sequences were aligned using ClustalW version 1.83 software and analyzed using MEGA6 version 6 software for Maximum likelihood analyses, and MrBayes version 3.1.2 software for Bayesian analysis.

**Table 1.** GenBank accession numbers for leech sequences used in the phylogenetic analysis of *Placobdelloides*.

Taxon	GenBank accession numbers	
	COI	ND1
Ingroup		
AN000016CE	MH777419	MH777409
AN000017CE	MH777420	MH777413
AN000018CE	MH777418	MH777411
AN000021CE	MH777415	MH777412
AN000022CE	MH777417	MH777410
AN000023CE	MH777416	MH777414
*Placobdelloides jaegerskioeldi*	AY962463	AY962450
*Placobdelloides multistriata*	DQ414338	DQ414383
*Placobdelloides siamensis*	AY962449	AY962462
Outgroup		
*Alboglossiphonia heteroclite*	AF116016	AY047339
*Alboglossiphonia quadrata*	AY962455	AY962441
*Alboglossiphonia weberi*	AY962453	AY962440
*Batracobdelloides tricarinata*	AY962457	AY962445
*Glossiphonia baicalensis*	AY047329	AY047355
*Glossiphonia complanate*	MF458715	AY047345
*Glossiphonia concolor*	AY962458	AY962446
*Glossiphonia elegans*	AY047322	AY047335
*Glossiphonia verrucata*	AY962459	AY962447
*Helobdella fusca*	AF329038	AF329061
*Helobdella robusta*	MF067148	MF067201
*Hemiclepsis marginata*	AF003259	AY047336
*Hirudo medicinalis*	HQ333517	KU672396
*Marsupiobdella africana*	AF116015	AY047347
*Placobdella montifera*	MF067129	MF067212
*Placobdella pediculate*	MF067121	MF067222
*Theromyzon bifarium*	AY047330	AY047356
*Theromyzon tessulatum*	AY047318	AY047338

**Table 2.** Pairwise *p*-distance values of the COI-ND1 gene within and among 20 species of the Glossiphoniidae family, *Hirudo medicinalis* and the sequences (AN000016CE, AN000017CE, AN000018CE, AN000021CE, AN000022CE and AN000023CE) identified in this study.

	1	2	3	4	5	6	7	8	9	10	11	12	13	14	15	16	17	18	19	20	21
*Hirudo medicinalis*																					
*Glossiphonia concolor*	0.802																				
*Alboglossiphonia quadrata*	0.536	0.457																			
*Alboglossiphonia heteroclite*	0.569	0.538	0.288																		
*Alboglossiphonia weberi*	0.518	0.428	0.205	0.193																	
*Helobdella robusta*	0.615	0.498	0.270	0.325	0.260																
*Helobdella fusca*	0.599	0.525	0.252	0.330	0.254	0.185															
*Placobdelloides siamensis*	0.514	0.435	0.249	0.294	0.217	0.253	0.269														
These studied sequences	0.512 0.561	0.431 0.457	0.245 0.288	0.294 0.347	0.218 0.262	0.250 0.295	0.269 0.310	0.007 0.075	0.002 0.081												
*Placobdella montifera*	0.554	0.473	0.263	0.336	0.257	0.218	0.239	0.215	0.217 0.260												
*Placobdella pediculate*	0.531	0.482	0.257	0.317	0.233	0.259	0.260	0.222	0.224 0.260	0.198											
*Glossiphonia elegans*	0.507	0.340	0.222	0.262	0.175	0.259	0.276	0.202	0.202 0.247	0.235	0.220										
*Glossiphonia verrucata*	0.538	0.336	0.226	0.302	0.197	0.276	0.297	0.220	0.218 0.257	0.256	0.253	0.123									
*Glossiphonia baicalensis*	0.509	0.327	0.211	0.272	0.185	0.256	0.269	0.205	0.201 0.249	0.224	0.231	0.108	0.091								
*Glossiphonia complanate*	0.494	0.339	0.217	0.273	0.179	0.257	0.272	0.189	0.189 0.229	0.232	0.220	0.101	0.107	0.091							
*Marsupiobdella africana*	0.522	0.466	0.262	0.294	0.229	0.245	0.262	0.191	0.193 0.243	0.226	0.229	0.214	0.242	0.222	0.220						
*Batracobdelloides tricarinata*	0.554	0.452	0.257	0.317	0.229	0.260	0.240	0.202	0.203 0.238	0.215	0.236	0.217	0.225	0.218	0.195	0.206					
*Hemiclepsis marginata*	0.520	0.455	0.224	0.317	0.225	0.272	0.256	0.194	0.193 0.238	0.222	0.226	0.214	0.221	0.199	0.210	0.218	0.199				
*Placobdelloides multistriatus*	0.500	0.431	0.214	0.275	0.187	0.232	0.225	0.171	0.171 0.218	0.190	0.189	0.199	0.197	0.177	0.175	0.177	0.170	0.181			
*Placobdelloides jaegerskioeldi*	0.507	0.424	0.235	0.291	0.198	0.254	0.263	0.166	0.165 0.205	0.202	0.210	0.191	0.190	0.186	0.182	0.197	0.194	0.181	0.142		
*Theromyzon bifarium*	0.518	0.414	0.210	0.292	0.191	0.239	0.245	0.158	0.156 0.199	0.191	0.191	0.178	0.179	0.179	0.166	0.186	0.185	0.162	0.144	0.152	
*Theromyzon tessulatum*	0.522	0.419	0.214	0.294	0.194	0.242	0.249	0.160	0.160 0.201	0.194	0.193	0.179	0.183	0.181	0.169	0.189	0.186	0.166	0.144	0.155	0.003

Maximum likelihood analyses consisted of 2000 replicates of tree search with 25 initial GAMMA rate categories and final optimization using four GAMMA shape categories; bootstrap values were calculated using 2000 pseudoreplicates of the rapid bootstrap algorithm. And Bayesian analysis was run for 2 million generations with tree sampled every 100 generations, with the general time reversible (GTR) model and GAMMA distribution of nucleotide rates for all partitions. Burn-in was set to 10%.

## Results

### Description

Specimens were determined to belong to *Placobdelloides* 1986 and were characterized as triannulate in the mid-body, with one pair of eyes on somite III, a terminal mouth pore, a crop with seven pairs of gastric ceca, a gonopore between two annuli, a caudal sucker slightly over the half body width, and cocoons and eggs attached directly to both the ventral surface of an adult and to the substrate [1, 8, 20].

### *Placobdelloides siamensis* (Oka, 1917) Sawyer, 1986

Syns. *Hemiclepsis siamensis* Oka, 1917; *Placobdelloides siamensis* (Oka, 1917) Sawyer, 1986.

### Taxonomic summary

*Type host*: Black Marsh Turtle, *Siebenrockiella crassicollis* (Gray, 1831) Mertens *et al*., 1934

*Additional hosts*: Southeast Asian Box Turtle (*Cuora amboinensis*, Daudin, 1802); Yellow-headed Temple Turtle (*Heosemys annandalii*, Boulenger, 1903); Mekong Snail-eating Turtle (*Malayemys subtrijuga*, Schlegel and Müller, 1845, Lindholm, 1931); Malayan Snail-eating Turtle (*M. Macrocephala*, Gray, 1859, Brophy, 2004); Khorat Snail-eating Turtle (*M. Khoratensis,* Ihlow, Vamberger, Flecks, Hartmann, Cota, Makchai, Meewattana, Dawson, Kheng, Rödder and Fritz 2016) (this study).

*Additional locality*: Kasetsart University, Bangkok Province (13°50′53.6″ N, 100°33′47.3″ E), Phraya Suren Temple market, Bangkok Province (13°52′23.6″ N, 100°42′03.6″ E), Udon Thani District, Udon Thani Province (17°36′55.5″ N, 102°81’42.7″ E)

*Site of infection*: external surfaces (carapace, head, neck, axilla, groin and tail), including inside mouth (Fig. 5)

*Type locality*: Lampam District, Patalung Province, Thailand.

*Specimen deposited:* Zoological Museum Kasetsart University (ZMKU), Department of Zoology, Faculty of Science, Kasetsart University, Bangkok, Thailand, ZMKU-ANN-0001-5.

### External morphology

**Figure 1.**
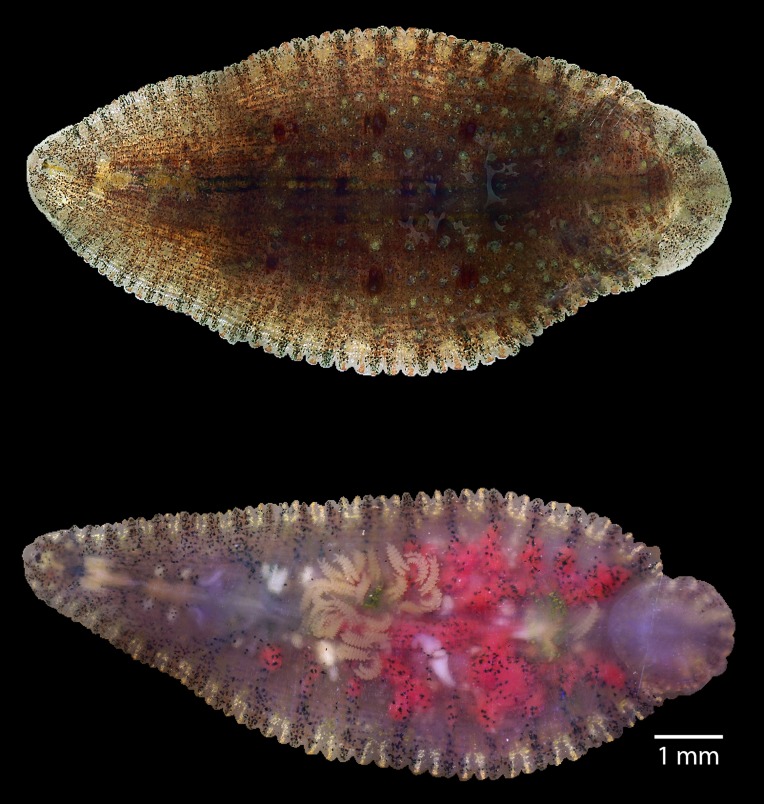
Dorsal surface (upper) and ventral surface (lower) of *Placobdelloides siamensis*. Ventral surface showing numerous young leeches.

Body of mature *P. siamensis* an elongated oval, 25 mm in length, maximum head and body width 2.35 and 5.57 mm, respectively (Fig. 1). Entire dorsal surface rough, with numerous well-developed 5–9 longitudinal rod papillae present. No regular arrangement pattern, except a row of papillae on each side of the median line. Ventral surface entirely smooth.

**Figure 2.**
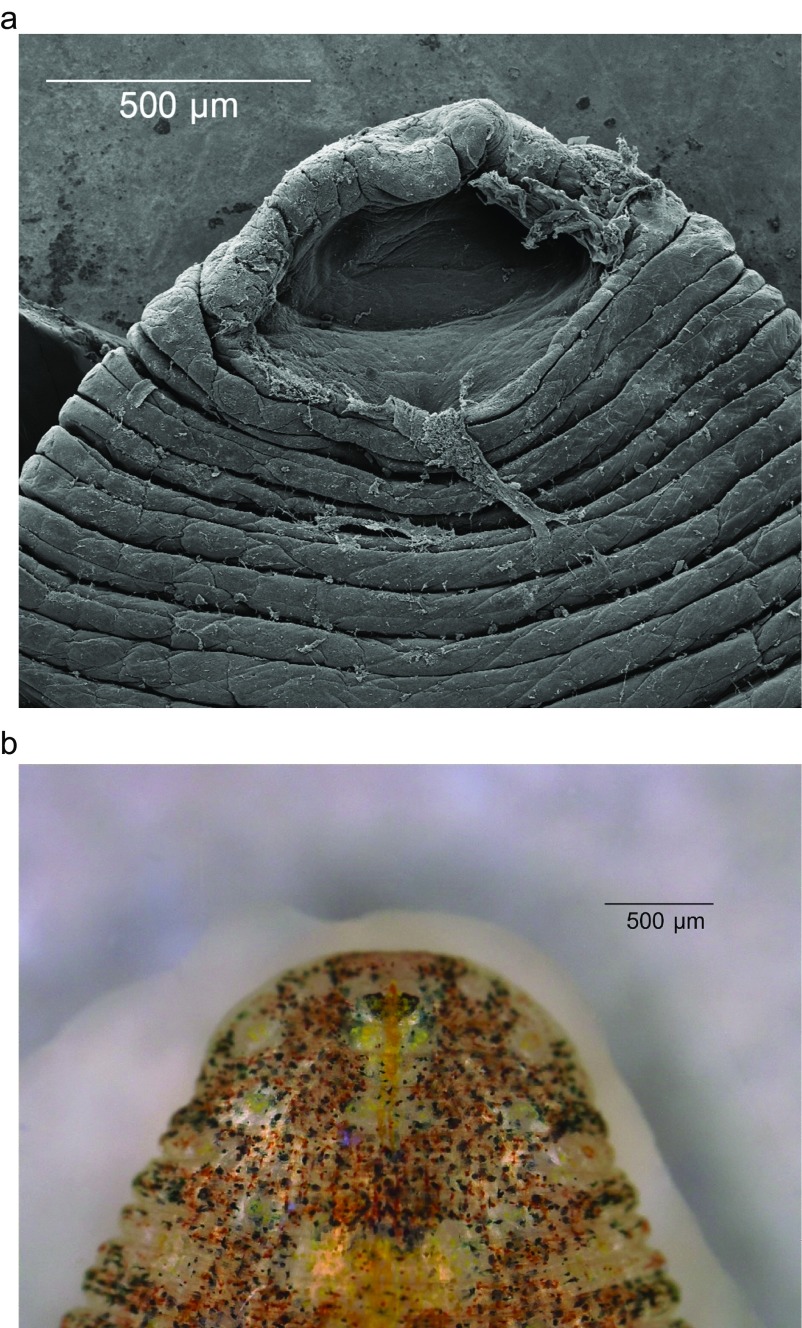
Anterior part of *Placobdelloides siamensis*. (A) Scanning electron microscope (SEM) showing oral sucker, numerous pits inside and mouth at terminal lip, (B) Dorsal surface showing eye placement at somite III. The dorsal color is uniformly brownish-gray, with randomly distributed dark brown, yellow, and green spots. The dorsal median line is yellowish, between four pairs of scarlet ovals. On the margin, brown, yellow, and green spots are present along the caudal sucker. The ventral surface is transparent.

**Figure 3.**
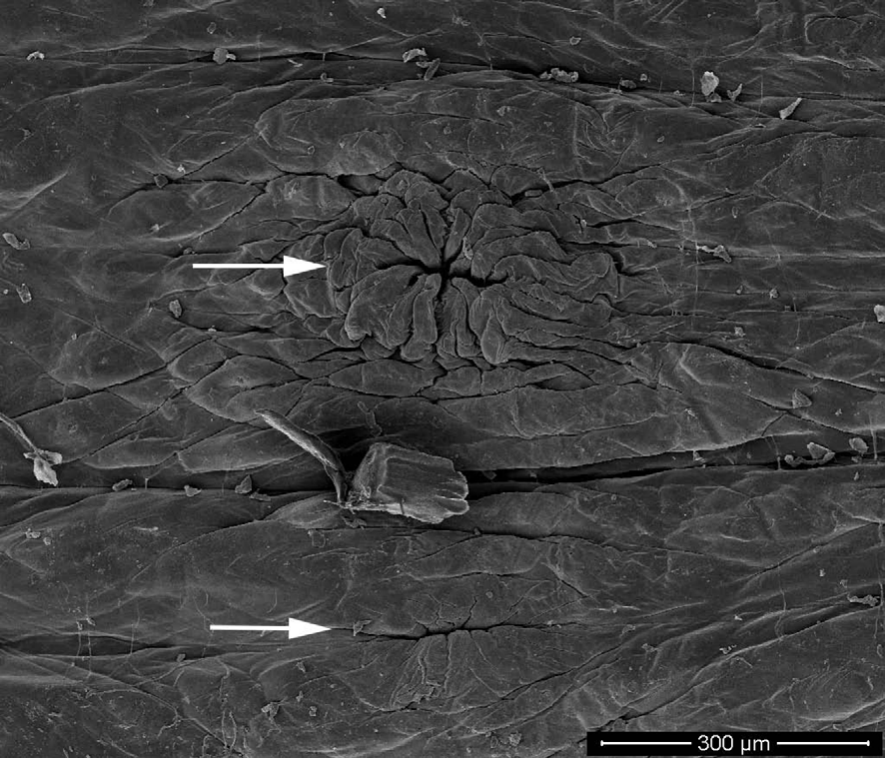
Scanning electron micrograph of the ventral surface of *Placobdelloides siamensis* showing the arrangement of gonopores. Upper image is the male gonopore and lower is female.

Oral sucker small (average 1.86 mm diameter), with mouth opening at terminal portion and numerous pits inside (Fig. 2A). One pair of eyes, located on dorsal surface of somite III (Fig. 2B). Eyes touch. Male genital pore situated in furrow between annuli 22 (3rd ring of somite X) and 23 (1st ring of somite XI) (Fig. 3). Female pore lies in furrow of somite XI between annuli 24 (1st ring) and 25 (2nd ring). Two annuli separate the gonopores, and anus separates last annulus and caudal sucker. Caudal sucker over half of body width (average 3.00 mm in diameter), no pits.

### Internal anatomy

**Figure 4.**
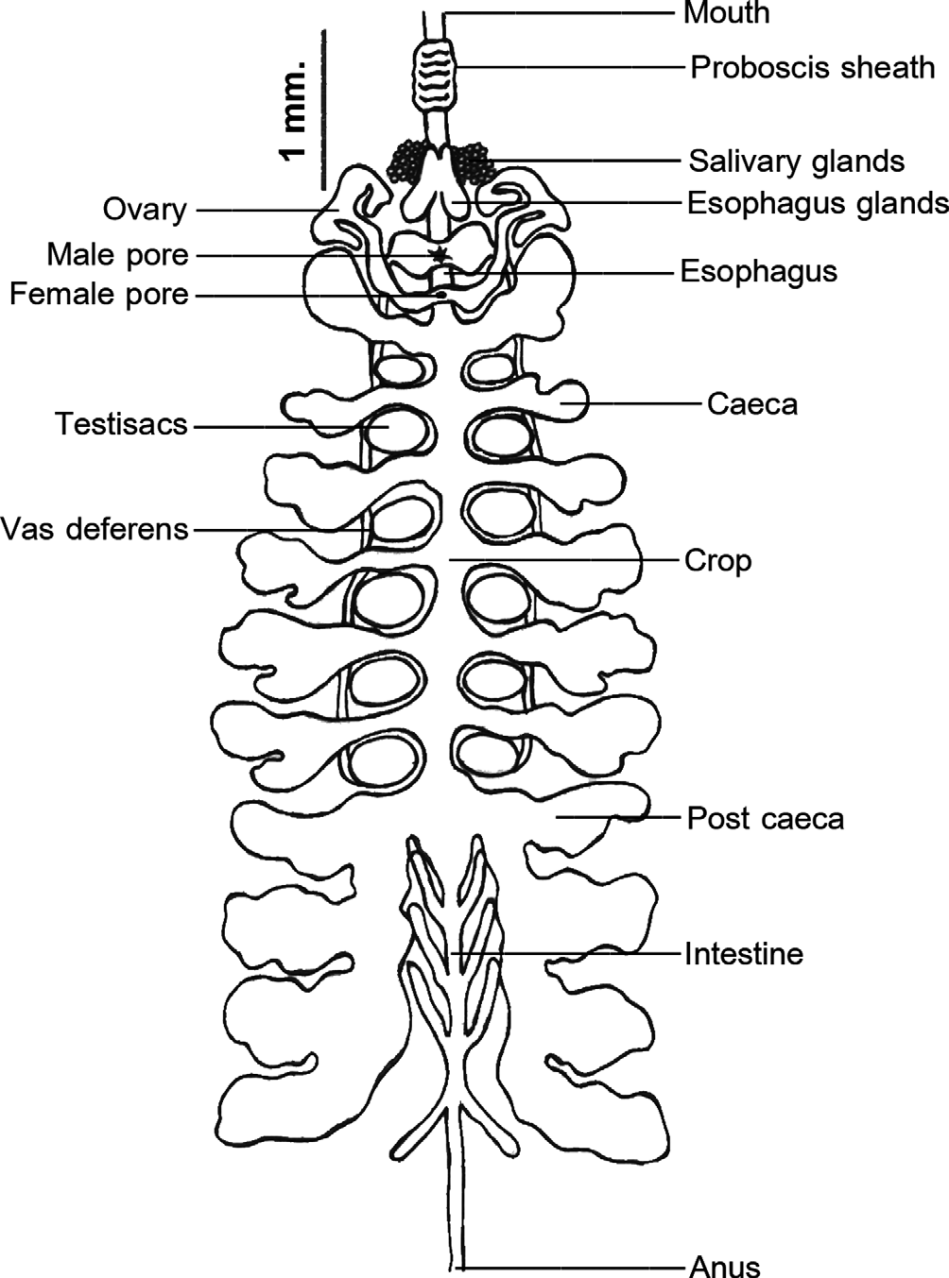
Internal anatomy of *Placobdelloides siamensis*.

Blunt elongated cylindrical proboscis from terminal lip of oral sucker to posterior proboscis sheath, followed by elongated esophagus, to which discrete compact bunch of salivary glands and diverticulate esophagus glands open. Seven pairs of diverticulated crop ceca, with last pair extending posteriorly and diverticulated into four sections. Four pairs of simple saccular intestinal, with last pair extending posteriorly to simple rectum and anus. Anus opens on dorsal surface behind the last annulus (Fig. 4).

**Figure 5.**
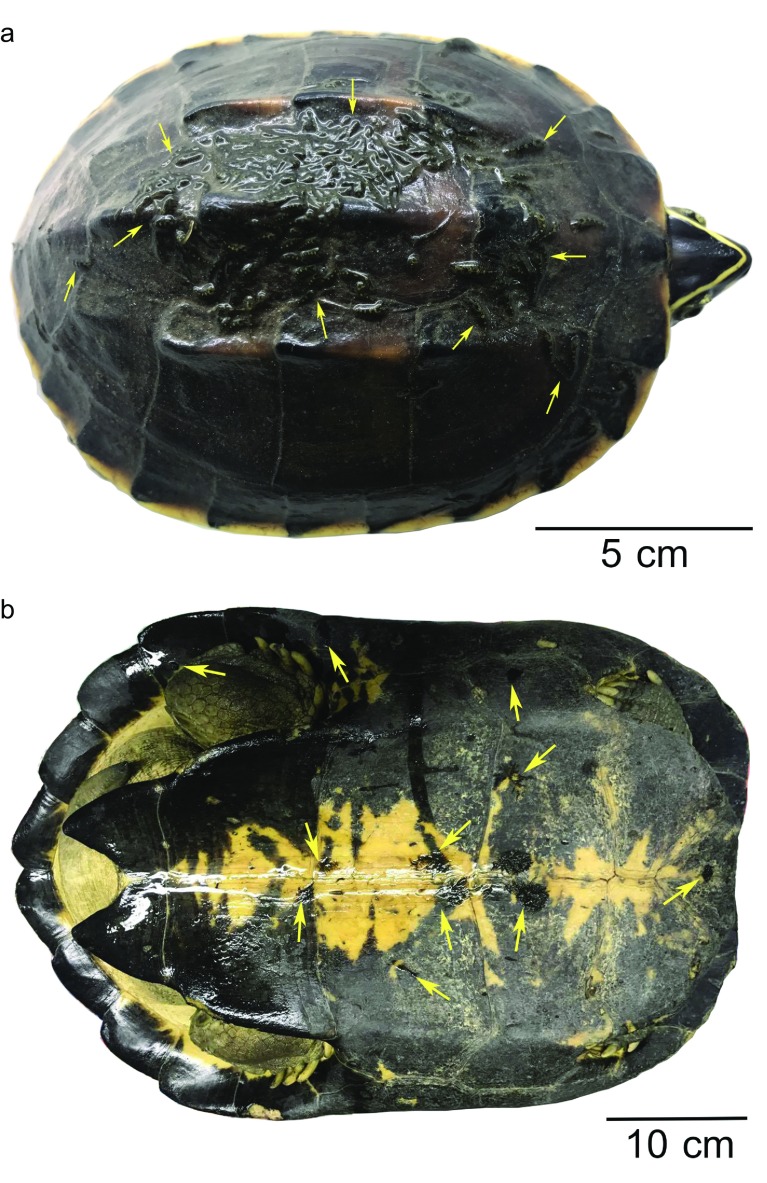
Parasitism of *Placobdelloides siamensis* on (A) the carapace of *Malayemys subtrijuga* and (B) plastron of *Heosemys annandalii*.

Male genital pore opens to glasses shaped sac, posteriorly to the diverticulate esophagus, and diverts to vas deferens on each side. Vas deferens posterior, opens to six pairs of curly rounded testes, each located close to the distal edge of each crop branch. Female pore situated posteriorly to male genital pore, opens to a sac which continues into elongated ovary on each side. Typically, ovaries coiled anteriorly the first crop caeca; however, after mating and egg producing, ovaries are elongated posteriorly from numerous eggs inside them.

### Molecular analysis

**Figure 6.**
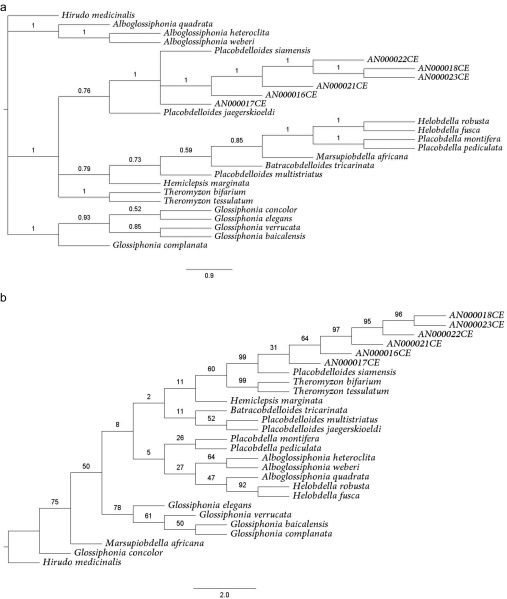
Phylogeny analysis of the COI-ND1 gene of glossiphoniid leeches (Table 2): the upper diagram is the Bayesian analysis; lower is the maximum likelihood analysis.

The Bayesian trees of the *COI–ND1* genes of the Glossiphoniid leeches had high probability support values for the monophyly of AN000016CE, AN000017CE, AN000018CE, AN000021CE, AN000022CE, AN000023CE, *P. siamensis*, and *P. Jaegerskioeldi*, but also revealed non-monophyly of *P. multistriatus* (Fig. 6). The maximum-likelihood trees also yielded monophyletic groups, similar to the Bayesian trees, but the data were different for *P. jaegerskioeldi* and *P. multistriatus*. The barcoding results also confirmed that AN000016CE, AN000017CE, AN000018CE, AN000021CE, AN000022CE, and AN000023CE were *P. siamensis*.

## Discussion

This is the first record of *P. siamensis* from *C. amboinensis, H. annandalii, M. macrocephala*, *M. subtrijuga*, and *M. khoratensis*. This leech has currently been recorded from six species of turtles from the same family (Geoemydidae).

In addition, this is the first record of *P. siamensis* from Udon Thani (northeastern province) in Thailand. The leech was previously reported only in Patalung (southern province) and Bangkok (central province) in Thailand, as well as in Nanking, China [20, 21]. Even though this leech has been shown to feed on numerous Geoemydidae turtles, as mentioned above, there have been no records that it is able to feed on other families, as found in this study. Moreover, it tends to spread throughout Thailand following its host distributions.

The morphological characters that separated this *P. siamensis* from it congeners are one pair of eyes; spines at the proboscis subterminal; mouth terminal on oral sucker; absent plaque in neck region; gonopores located in furrow and separated by two annuli; distinctly triannulated mid-body segments; crop with seven pairs and branched ceca; caudal sucker slightly over half maximum body width; and strongly dorsal papillae. The specimens collected in this study almost matched the description of *P. siamensis* (in alcohol) by Annandale [1], except the male and female gonopores. The male gonopore had been described as situated between annuli 25 and 26 (somite XI and XII), while it was situated between annuli 22 and 23 (somite X and XI) in this study.

The female gonopore had been described as situated between annuli 27 and 28 (somite XII), while it was situated between annuli 24 and 25 (somite XI) in this study. Since Annandale [1] used preserved specimens, no information on coloration in life was provided. The report by Siddall *et al*. [21] on the sequencing of *P. siamensis* from Wat Bovorn did not describe morphological details, nor the host. Collection of a large number of live leeches in this study allowed us to describe the external coloration and morphology.

All specimens examined were mature, with developed testisacs and ovisacs present. Some specimens carried cocoons or young on the posterior ventral surface, as in the original description. In this study, some cocoons were attached to the substrate. The number of eggs was documented to be between 173 and 412 per individual in 14 specimens.

In the original description, Annandale [1] hesitated to classify this species into *Hemiclepsis* or *Placobdella* because of the oval shape, one pair of eyes, a pattern of numerous irregular papillae, gonopores separated by two annuli, and cocoons carried on the ventral side. Finally, it was placed in *Hemiclepsis* based on the distinctly posterior position of the mouth, although *Hemiclepsis* is characterized by 9–11 pairs of crop ceca, 2–3 pairs of eyes, gonopores separated by two annuli, and the attachment of cocoons to the substrate [20].

Sawyer [20] subsequently moved *H. siamensis* to *P. siamensis* because of the following similarities: one pair of eyes, an esophageal organ, seven pairs of crop ceca, a terminal mouth pore, gonopores separated by two annuli, and a combination of the parental care behavior, either carrying cocoons on the ventral surface or attached to the substrate.

Although Annandale [1] and Sawyer [20] clearly described the morphology of *P. siamensis*, this study provides a more complete overview of the morphological structure, coloration pattern, and parental care behaviors of *P. siamensis*, thus leading to a better understanding of its actual phylogenetic position within glossiphoniid leeches.
